# Associations Between Engagement With an Online Health Community and Changes in Patient Activation and Health Care Utilization: Longitudinal Web-Based Survey

**DOI:** 10.2196/13477

**Published:** 2019-08-29

**Authors:** Ruth E Costello, Amrutha Anand, Matt Jameson Evans, William G Dixon

**Affiliations:** 1 Centre for Epidemiology Versus Arthritis Manchester Academic Health Science Centre The University of Manchester Manchester United Kingdom; 2 HealthUnlocked (Everything Unlocked Ltd) London United Kingdom; 3 NIHR Manchester Biomedical Research Centre Manchester University NHS Foundation Trust Manchester United Kingdom

**Keywords:** self-management, chronic disease, health information exchanges, digital health, peer support, peer-to-peer support, online support groups, internet

## Abstract

**Background:**

Participation in online health communities (OHCs) is a popular trend in the United Kingdom. However, so far, no evidence exists to indicate an association between participation in OHCs and improved health outcomes.

**Objective:**

This study aimed to (1) determine changes in patient activation over 3 months in new users of an OHC, (2) describe patterns of engagement with an OHC, (3) examine whether patients’ characteristics at baseline were associated with subsequent patterns of engagement, and (4) determine if patterns of engagement during the 3 months were associated with changes in patient activation, health care utilization, and health status.

**Methods:**

Active new OHC users on HealthUnlocked (HU) were surveyed to measure demographics, levels of patient activation (describing a person’s confidence in managing their own health; scale 0-100 with 4 categories), health care utilization, and health status using a Web-based survey at baseline and 3 months. Patient activation at baseline and 3 months was compared (aim 1). Alongside, for a sample of HU users and survey responders, daily OHC website usage data were automatically captured. This was used to identify clusters of engagement with HU (aim 2). For survey responders, baseline characteristics, patient activation, health care utilization, and health status were compared at baseline and 3 months, overall, and between engagement clusters using t tests and chi-square tests (aims 3 and 4).

**Results:**

In 329 people who completed both surveys, baseline activation was most frequently level 3, described as taking action but still lacking confidence. At follow-up, a change of 2.6 points was seen, with the greatest change seen in those at lowest baseline activation levels. In addition, 4 clusters of engagement were identified: low, medium, high, and very high, who were active on HU for a mean of 4, 12, 29, and 59 days, respectively. Survey responders were more commonly high or very high engagers. Baseline activation was highest in low and very high engagers. Overall activation increased over time in all engagement groups. Very high engagers had the greatest improvement in activation (5 points), although the average change was not above what is considered clinically meaningful for any group. Fewer accident and emergency visits were seen at follow-up in those with higher engagement, although this trend was not seen for other health care utilization measures. There was no change in health status at 3 months.

**Conclusions:**

This observational study provides some insight into how patterns of engagement with OHCs are associated with changes in patient activation, health care utilization, and health status. Over 3 months, overall, the change in activation was not clinically significant, and there were some indications that OHCs may be of benefit to particular groups. However, the study limitations prevent firm conclusions about causal relationships.

## Introduction

The internet is used by more than 90% of the population in the United Kingdom, according to a national survey in 2017. Of those who had used the internet in the last 3 months, 53% of people had looked for health-related information [1]. In addition to accessing websites from trusted organizations, such as the National Health Service, and patient organizations or charities, for example, Cancer Research UK, people are increasingly using peer-to-peer online health communities (OHCs). OHCs are forums where people with specific conditions can share information and experiences with other people with the same condition through peer support and discussion. A survey of US adults found that 1 in 4 people read or watched commentary of another person’s experiences of health or medical issues on the Web [2]. Learning about other people’s experiences online may have a number of impacts, ranging from finding information and feeling supported to affecting behavior [3].

One way that OHCs may be particularly useful is in helping with self-management of chronic diseases. Studies analyzing OHC content have found that conversations may support self-efficacy [4,5]. A cross-sectional survey of users of OHCs for 3 different conditions (breast cancer, fibromyalgia, and arthritis) found that 74% felt they now had *the right knowledge to manage their illness* after participation in an OHC [6]. One might therefore argue that accessing online information and peer support would lead to empowerment, improved coping, reduced uncertainty, and potentially even reduced health care utilization [7]. Studies have found OHC use to be associated with increased empowerment [6,8,9] with themes of feeling better informed and social support [6-8,10,11]. Conversely, there are arguments that information in OHCs may not be reliable or accurate, and peer support, rather than information from health care professionals, may amplify anxiety [12,13].

Patient activation is defined as a person’s knowledge, skills, and confidence in managing their health or chronic condition [14]. As a concept, patient activation differs slightly from empowerment; it builds on self-efficacy and aims to capture the development of a patient’s engagement with managing their own health, from believing they have an active role to having the confidence to self-manage their own health when under stress [15]. The measure has been shown to be reliable and valid [15] in a variety of countries and populations [16-21]. Higher activation has been associated with positive health behaviors such as attending screenings and eating 5 or more fruits and vegetables per day [22]. Studies have shown that those with lower activation scores are more likely to be hospitalized and more likely to visit an accident and emergency department [23]. At present, it remains uncertain as to whether interventions to improve disease knowledge can improve activation and in turn lead to improved behaviors and health outcomes. Studies investigating similar concepts such as empowerment and self-efficacy are frequently cross-sectional [5,6]. To date, only 1 study has examined the relationship between OHCs and activation specifically. In this study, experienced users of an OHC had higher activation scores than new users, and activation scores in both groups increased after 3 months’ OHC use, with higher scores in those who self-reported using the site more frequently [24]. This study did not investigate subsequent health care utilization.

Studying the relationship between engagement with OHCs and patient activation and health outcomes is complex because of (1) confounding by indication (where people accessing the platform are inherently different from those who do not, which in turn affects their probability of the outcomes of interest) [25], (2) natural changes in health care utilization at different stages of disease (eg, general practitioner [GP] visits may naturally be higher around the time of diagnosis than in subsequent periods), and (3) challenges in quantifying the *exposure* of OHC engagement [26]. However, because website visits leave digital traces, it is possible to measure how commonly people interact with a site, thereby allowing the relationship between different engagement patterns and outcomes to be studied.

This study aimed to (1) determine change in activation over 3 months in new users of an OHC, (2) describe patterns of engagement with an OHC, (3) examine whether patient characteristics at baseline were associated with subsequent patterns of engagement, and (4) determine if patterns of engagement during the 3 months were associated with changes in patient activation, health care utilization, and health status.

## Methods

### Setting

HealthUnlocked (HU) is a host to multiple OHCs with more than 4.5 million visitors each month. HU has more than 700 communities for a variety of health conditions as well as hosting communities to support aspects of well-being, for example, weight loss and healthy eating [27]. These OHCs are built in collaboration with patient organizations, who moderate the communities, to ensure safety of its users and verify credibility of content shared. Often, expert users with no formal association with a patient organization volunteer to moderate communities focused on health and well-being.

Once registered on the platform, a user can choose to follow communities relevant to their health interest and post questions, updates, or any information that they wish to share, or reply to previous posts from other users. In addition to text, users can post images on these OHCs. Users can also like other posts or follow other users to build a network around them. An exemplar post is included in Multimedia Appendix 1.

### Data

This study used data from a survey (Multimedia Appendix 2) designed and run by HU in conjunction with a research team at King’s Health Economics to understand health and economic outcomes in new users of HU. The anonymized survey data were sent to the University of Manchester after the survey ended, and this analysis was designed to meet our study aims.

### Population

HU is freely available to the public, and people sign up with their email address and password. Those who signed up, followed at least one of the communities included in this study, and were active on the website between 48 and 72 hours after signing up were eligible for the study and were emailed the survey. People who completed the baseline survey were sent a follow-up survey 3 months later. Reminder emails for both surveys were sent 2 days after the original email. The survey started in September 2016 and continued until the sample size reached at least 300. This was based on the study having 90% power to identify a mean difference of 3 between baseline and follow-up in patient activation score.

### Survey

The survey asked about demographics (eg, age, gender, occupation, education, and ethnicity); information about health: main diagnosis (collected as free text and verified against community group followed); disease duration in response to the question “How long since you were diagnosed with the condition?” with the options less than a year ago, 1 to 3 years, 4 to 6 years, 7 to 9 years, and 10 years or more; and comorbidities in response to the question “Do you currently have other long-term concerns in addition to your diagnosis?” with the options No, Yes I have 1 more, Yes I have 2 more, and Yes I have more than 3. Patient activation was measured using the Patient Activation Measure (PAM). The measure contains 13 statements where respondents indicate whether they strongly agree (4 points), agree (3 points), disagree (2 points), or strongly disagree (1 point) with each of the statements [15]. Using a standardized table, these scores were converted to a score out of 100 where a higher score indicates a person showing greater activation. People with a score of 100 at baseline or follow-up were removed from the cohort during analysis as a score of 100 is considered implausible (Personal communication, C Delaney, 2018). PAM score was then converted to 4 levels of activation, as defined by the authors: level 1 (PAM score <47): overwhelmed and passive in managing their own health; level 2 (PAM score 47.1-55.1): lack of knowledge and confidence; level 3 (PAM score 55.2-72.4): taking action but still lacking confidence; and level 4 (PAM score 72.5-100): have adopted good health behaviors but may have problems when under stress [15]. Health status was measured using the EuroQol-5D (EQ-5D), which contained 5 questions about mobility, self-care, usual activities, pain, and depression or anxiety. Each question was scored between 1 and 5, and the score for each of these questions was weighted using a value set for the UK population and a single score created with the anchors 0 and 1 [28]. The health care utilization questionnaire asked, “In the last 3 months, roughly how many times have you visited the following healthcare services?”: GP, outpatient clinic, primary care nurse, accident & emergency (A&E) with the options never, 1 to 3 times, 4 to 6 times, and more than 6 times, and “In the last 3 months, roughly how many days have you spent admitted in a hospital?”: a free-text box allowed respondents to indicate the number of days. For the first question, categories 4 to 6 times and more than 6 times were combined because of small numbers. Number of days in hospital was categorized into none, 1 to 5 days, 6 to 10 days, and more than 10 days based on the spread of the data.

### HealthUnlocked Engagement

Engagement with HU was determined through the following measures, which are automatically captured daily: pages viewed, number of clicks anywhere on the website, number of community groups followed, number of users followed (subscribing to or following a community or user means posts from these communities or users will appear in the subscribers newsfeed), posts liked, written comments, and primary posts (starting a post) for each user. A daily count of each engagement measure was provided by HU for all people who completed both baseline and follow-up surveys and a random sample of 336 people who completed only the baseline survey and a random sample of 337 who completed neither survey.

### Analysis

Mean PAM scores at baseline and follow-up were compared using a *t* test. The proportion of people at each PAM level at baseline and follow-up were compared. Users were then grouped into clusters based on their daily HU engagement data. First, for each day, a person was flagged as having engaged with HU on that day if any of the HU engagement measure counts were not zero. As this was time series data, a first-order Markov Mixture model with an expected maximization algorithm was used to identify clusters [29]. First, the model identified the states of engagement each day, with 3 latent states assumed: high engagement, low engagement, or disengaged. Everybody started at high engagement, and disengagement was assumed to be an *absorbing state* after which there would be no further engagement. People were then clustered based on transitional probabilities of changing engagement state. The optimum number of cluster groups was identified using the elbow method [30]. Baseline characteristics were compared among cluster groups, and the mean number of days of engagement for each cluster group was reported.

Patient activation score, EQ-5D score, and health care utilization at baseline and follow-up were compared between cluster groups. Health care utilization measures were reduced to binary measures of whether participants had any visits in the last 3 months because of low numbers of people with a high number of visits. Box and whisker plots were used to show the distribution of PAM at baseline and follow-up. Kruskal-Wallis tests checked if there was a statistically significant difference in PAM scores between cluster groups. The proportion with a change of PAM score by more than 5 points (a suggested clinically meaningful difference [14]) was reported by engagement cluster. PAM score was then categorized into levels and compared at baseline and follow-up, with the proportions where PAM had increased, remained stable, and reduced reported by cluster group. EQ-5D scores were compared at baseline and follow-up, and a *t* test was used to see if there was a statistically significant difference. The percentage of people with each type of health care visit at baseline and follow-up for each cluster group was compared using chi-square tests.

### Ethical Approval

As this survey was service evaluation conducted by HU and King’s Health Economics, NHS ethical approval was not required. Ethical approval for the analysis was confirmed as not required by the University of Manchester’s ethics committee, as the data were already collected and were anonymized.

## Results

### Survey Response

The survey was sent to 9469 people; 990 people completed the baseline survey, of whom 329 completed the follow-up survey and had HU usage data available. Of those who completed the follow-up survey, 78.5% (258/329) were aged 50 years or older, 76.6% (252/329) were female, and 93.0% (305/328) were white. 87 (26.4%) were from musculoskeletal community groups (fibromyalgia, lupus, rheumatoid arthritis, polymyalgia rheumatica and giant cell arteritis, pain), and 67 (20.4%) were from endocrine (diabetes and thyroid) community groups (Table 1).

### Patient Activation

There were 15 people with a PAM score of 100 at baseline, follow-up, or both; therefore, change in PAM score is reported for only 314 people. For those who completed both surveys, the mean PAM scores at baseline and follow-up were 60.2 and 62.8, respectively, a statistically significant difference of 2.6 points (standard deviation: 8.4 points, *P*<.001). When stratified by baseline PAM level (1 and 2 vs 3 and 4), those at levels 1 and 2 had a statistically significant 5.5-point increase (95% CI 4.1-6.8; *P*<.001). Those at levels 3 and 4 had a nonsignificant 1.1-point increase (95% CI 2.3 to −0.05). When categorized into PAM levels, nearly half (49.4%, 155/314) were at level 3 (taking action but still lacking confidence), and overall, PAM level increased at follow-up (Multimedia Appendix 3).

### Engagement With HealthUnlocked

HU activity data were available across the 3-month period for all 329 participants who completed both surveys, random samples of 336 people who did not complete either survey, and 337 people who completed baseline only (total 1002).

Those who completed both surveys engaged with HU more frequently (median: 47 days) than those who only completed baseline (median: 24 days) or did not complete either survey (median: 9 days), although there was a wide spread in the number of people engaged with HU in each response group (Multimedia Appendix 4). In terms of people’s activities at visits to HU, 50.90% (510/1002) of participants posted at least once with a median 1 post per person (interquartile range [IQR]: 0-2) and a maximum of 84 posts over 3 months. A total of 15,431 comments were made by 63.07% (632/1002) of participants, with a median of 2 comments (IQR: 0-11) and a maximum of 1549 comments over 3 months. Those who did not complete either survey had fewer written posts, comments, and posts liked per visit to HU than those who completed both surveys, whereas those who completed the baseline survey only had similar numbers of written posts, comments, and likes per visit compared with those who completed both surveys.

**Figure 1:**
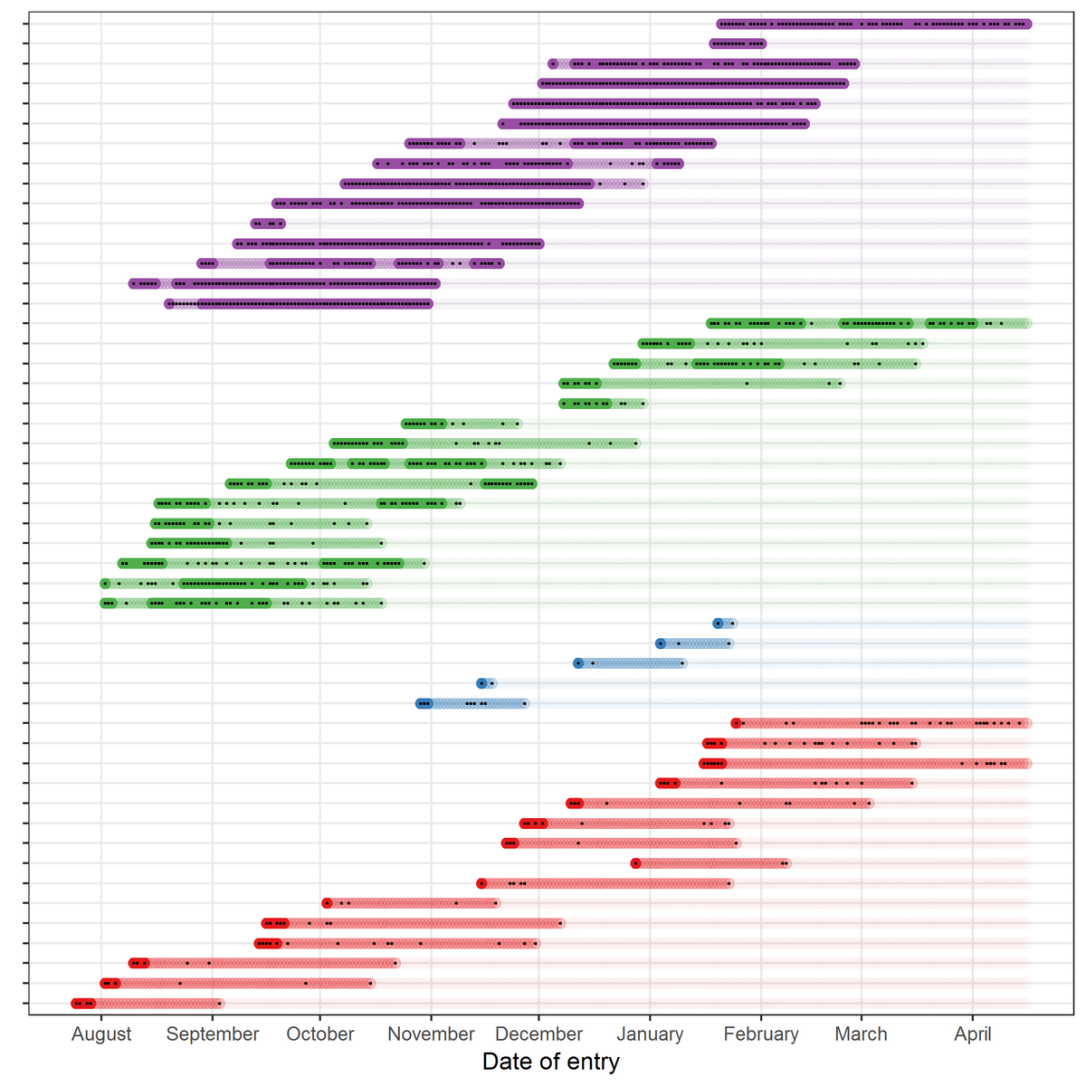
The number of days of engagement with HU and states of engagement by engagement cluster for a sample of all users (N=50). Each line represents a respondent, each dot represents a day the respondent engaged with the HU platform, the colors represent the engagement clusters, where blue indicates low engagers, red medium engagers, green high engagers, and purple very high engagers. The shading of the color represents the state of engagement where dark color indicates high engagement, and light color indicates low engagement. HU: HealthUnlocked.

The hidden Markov model identified 4 clusters: (1) low engagers: (142/1002, 14.17%) who were active on HU for a mean of 4.4 (SD 2.1) days before not visiting the platform further; (2) medium engagers: (216/1002, 21.55%) who were active on HU for a mean of 11.9 (SD 6.3) days; (3) high engagers: (338/1002, 33.72%) who were active for a mean of 29.1 (SD 13.0) days; and (4) very high engagers: (306/1002, 30.54%) who were active for a mean of 59.2 (SD 22.2) days (Figure 1).

The majority of those completing the follow-up survey were high or very high engagers (114/329, 34.7%; and 163/329, 49.5%, respectively). The mean number of active days was slightly higher in those who completed the survey (Table 2). All further results refer to those who completed both surveys.

**Table 1:** Baseline characteristics of respondents (N=329).

Characteristics		Values, n (%)
**Age (years)**		
	<40	26 (7.9)
	40-49	45 (13.7)
	50-59	94 (28.6)
	60-69	118 (35.9)
	>70	46 (14.0)
**Gender**		
	Male	77 (23.4)
	Female	252 (76.6)
**Ethnicity**		
	White	305 (93.0)
	Asian	6 (1.8)
	Black/African/Caribbean	7 (2.1)
	Hispanic/Latino	1 (0.3)
	Multiple ethnicities	9 (2.7)
	Missing	1 (0.3)
**Employment status**		
	Employed	97 (29.6)
	On sick leave, unable to work	37 (11.3)
	Retired	160 (48.8)
	Student	3 (0.9)
	Unemployed	31 (9.5)
	Missing	1 (0.3)
**Education**		
	Primary school	5 (1.5)
	Secondary school	156 (47.6)
	University degree	116 (35.4)
	Postgraduate degree	51 (15.5)
	Missing	1 (0.3)
**Comorbidities**		
	None	137 (42.0)
	1	103 (31.6)
	≥2	86 (26.4)
	Missing	3 (0.9)
**Community**		
	Cardiovascular	41 (12.5)
	Respiratory	12 (3.6)
	Cancers	34 (10.3)
	Mental health	7 (2.1)
	Digestive system	44 (13.4)
	Endocrine	67 (20.4)
	Genitourinary	16 (4.9)
	Musculoskeletal	87 (26.4)
	Nervous system	4 (1.2)
	Blood disorders	16 (4.9)
	Reproductive	1 (0.3)
**Time since diagnosis (years)**		
	<1	113 (35.1)
	1-6	105 (32.6)
	>7	104 (32.3)
	Missing	7 (2.1)

**Table 2:** Number of days of engagement with HealthUnlocked by cluster.

Engagement cluster	Both surveys completed (N=329)		Baseline survey completed (N=337)		No surveys completed (N=336)	
	n (%)	Active days, mean (SD)	n (%)	Active days, mean (SD)	n (%)	Active days, mean (SD)
Low	18 (5.4)	6 (2.4)	31 (9.1)	5 (3)	93 (27.6)	3 (3)
Medium	34 (10.3)	14.3 (6.5)	69 (20.4)	13 (10)	113 (33.6)	9 (7)
High	114 (34.6)	34.4 (12.9)	144 (42.7)	26.5 (17.5)	80 (23.8)	22 (17)
Very high	163 (49.5)	66.4 (15.8)	93 (27.5)	60 (28)	50 (14.8)	55 (60)

### Characteristics

The characteristics of each cluster of survey responders are shown in Table 3. High and very high engagers had a higher proportion of females (95/114, 83.3% and 124/163, 76.1%, respectively) compared with medium and low engagers (22/35, 64% and 11/18, 61%, respectively). Higher engagers had less comorbidity: 22.2% (n=36/162) of very high engagers had 2 or more comorbidities compared with 55.6% (n=10/18) of low engagers, and a shorter time since diagnosis: 39.0% (n=62/159) of very high engagers were diagnosed less than a year ago compared with 19% (n=3/16) of low engagers.

### Patient Activation by Engagement Cluster

**Figure 2:**
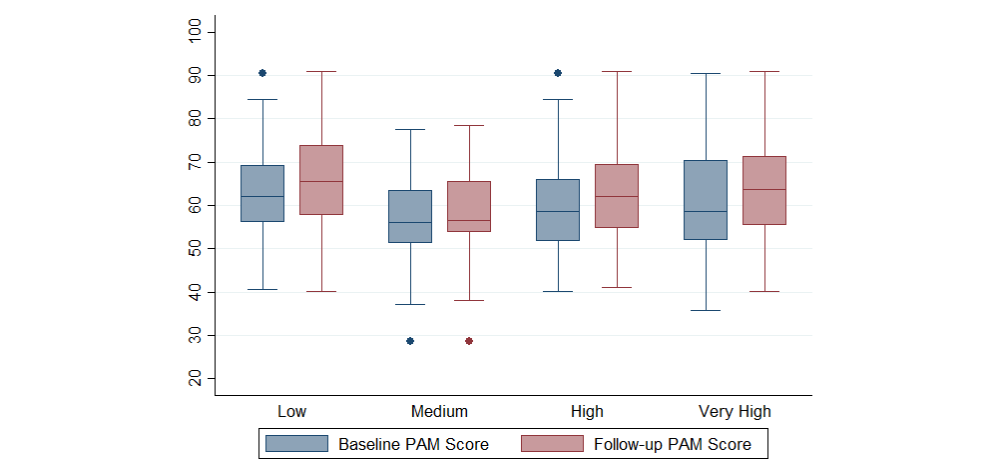
Box and whisker plot of PAM scores by engagement cluster (N=314). Box plots represent the median (central line), interquartile range (box), range, excluding outliers (whiskers), and outliers (dots) of the percentage of patients within each engagement cluster. PAM: Patient Activation Measure.

Figure 2 shows the distribution of PAM scores for each engagement cluster at baseline and follow-up. Median baseline PAM scores differed little across the 4 engagement groups with a difference of only 6 points from highest to lowest. PAM scores increased at follow-up in all engagement groups. Medium engagers had the lowest change of 0.5 points, and very high engagers had the highest change of 5.1 points.

When scores were categorized into PAM levels, 81% (13/16) of low engagers were at level 3 or 4 at baseline, increasing 94% (15/16) at follow-up. All other engagement clusters had around half at level 3 at baseline. Medium engagers had the highest proportion at level 2, and very high engagers had the highest proportion at level 4. At follow-up, the proportion at level 3 increased for all engagement clusters, and the only engagement cluster with an increased proportion at level 4 was the high engagement cluster (13.6% [15/110] at baseline vs 17.3% [19/110] at follow-up). Moreover, 22.2% (36/163) and 20.5% (33/163) of very high engagers were at level 4 at both baseline and follow-up, respectively (Table 4).

A PAM score increase of at least 5 points was seen in 35.0% (110/314) of respondents, with the highest proportion in the very high engagers (63/156, 40.4%) and lowest proportion in medium engagers (6/32, 18%). A PAM score decrease of at least 5 points was seen in 15.0% (47/314), with proportions similar across engagement clusters.

### Health Status

Respondents had a mean EQ-5D score of 0.69 at baseline and 0.70 at follow-up, where 1 indicates perfect health, and less than zero indicates a state worse than death. There was little difference in average health status between baseline and follow-up within the 4 engagement clusters, with a maximum mean change within groups of 0.02 units.

### Health Care Utilization

At baseline, 88.4% (283/320) of people visited their GP at least once and 37.5% (102/272) visited a primary care nurse at least once in the previous 3 months. 64.2% (190/296) people visited outpatients in the previous 3 months. 21.7% (62/286) visited A&E and 21.6% (71/328) were hospitalized in the previous 3 months. At follow-up, the proportion of people visiting a GP was slightly lower at 83.8% (268/320), and the proportion visiting a primary care nurse was similar to baseline. The proportion of people visiting outpatients or A&E or being hospitalized reduced, the biggest reduction being in A&E visits where only 12.6% (36/285) visited A&E at follow-up (Table 5). When stratified by engagement cluster, all engagement clusters had fewer people visiting a GP, outpatients, and being hospitalized at least once at follow-up, except medium engagers where the proportion of people visiting a GP remained the same. The proportion of people visiting a primary care nurse varied across engagement clusters with no clear pattern. The only statistically significant difference between engagement clusters was for A&E visits at follow-up, where there was a trend toward those with greater engagement with HU having a smaller proportion of people visiting A&E at follow-up. Low engagers had a 12% more people visiting A&E, and very high engagers had a 12.6% fewer people visiting A&E at follow-up (Table 5).

**Table 3:** Baseline characteristics of baseline and follow-up survey respondents by engagement cluster (N=329).

Baseline characteristic		Engagement cluster, n (%)			
		Low (n=18)	Medium (n=34)	High (n=114)	Very high (n=163)
**Age (years)**					
	<40	1 (5)	3 (8)	14 (12.3)	8 (4.9)
	40-49	3 (16)	7 (20)	17 (14.9)	18 (11.0)
	50-59	6 (33)	6 (17)	36 (31.6)	46 (28.2)
	60-69	6 (33)	15 (44)	33 (28.9)	64 (39.3)
	>70	2 (11)	3 (8)	14 (12.3)	27 (16.6)
**Gender**					
	Male	7 (38)	12 (35)	19 (16.7)	39 (23.9)
	Female	11 (61)	22 (64)	95 (83.3)	124 (76.1)
**Ethnicity**					
	White	17 (94)	32 (94)	101 (88.6)	155 (95.7)
	Asian	1 (5)	1 (2)	2 (1.8)	2 (1.2)
	Black/African/Caribbean	0 (0)	1 (2)	6 (0.0)	0 (0.6)
	Latino	0 (0)	0 (0)	0 (5.3)	1 (0.0)
	Multiple ethnicities	0 (0)	0 (0)	5 (4.4)	4 (2.5)
	Missing	0	0	0	1 (0.6)
**Employment status**					
	Employed	3 (16)	8 (23)	46 (40.4)	40 (24.7)
	On sick leave, unable to work	2 (11)	4 (11)	13 (11.4)	18 (11.1)
	Retired	9 (50)	17 (50)	44 3(8.6)	90 (55.6)
	Student	1 (5)	1 (2)	1 (0.9)	0 (0.0)
	Unemployed	3 (16)	4 (11)	10 (8.8)	14 (8.6)
	Missing	0	0	0	1 (0.6)
**Education**					
	Primary school	1 (5)	1 (2)	3 (2.6)	0 (0.0)
	Secondary school	10 (55)	19 (55)	54 (47.4)	73 (45.1)
	University degree	5 (27)	12 (35)	41 (36.0)	58 (35.8)
	Postgraduate degree	2 (11)	2 (5)	16 (14.0)	31 (19.1)
	Missing	0	0	0	1 (0.6)
**Comorbidities**					
	None	4 (22)	18 (52)	46 (41.1)	69 (42.6)
	1	4 (22)	5 (14)	37 (33.0)	57 (35.2)
	≥2	10 (55)	11 (32)	29 (25.9)	36 (22.2)
	Missing	0	0	2 (1.8)	1 (0.6)
**Community**					
	Cardiovascular	2 (11)	3 (8)	14 (12.3)	22 (13.5)
	Respiratory	1 (5)	1 (2)	4 (3.5)	6 (3.7)
	Cancers	2 (11)	4 (11)	8 (7.0)	22 (13.5)
	Mental health	5 (27)	1 (2)	2 (1.8)	2 (1.2)
	Digestive system	4 (22)	5 (14)	12 (10.5)	22 (13.5)
	Endocrine	4 (22)	7 (20)	21 (18.4)	35 (21.5)
	Genitourinary	5 (27)	2 (5)	11 (9.6)	3 (1.8)
	Musculoskeletal	20 (30)	10 (29)	34 (29.8)	39 (23.9)
	Nervous system	1 (1)	1 (2)	1 (0.9)	2 (1.2)
	Blood disorders	1 (1)	1 (1)	6 (5.3)	10 (6.1)
	Reproductive	0 (0)	1 (0)	1 (0.9)	0 (0.0)
**Time since diagnosis (years)**					
	<1	3 (18)	8 (23)	40 (35.4)	62 (39.0)
	1-6	5 (31)	12 (35)	32 (28.3)	56 (35.2)
	≥7	8 (50)	14 (41)	41 (36.3)	41 (25.8)
	Missing	2 (11.1)	0	1 (0.9)	4 (2.5)

**Table 4:** Baseline and follow-up Patient Activation Measure level by engagement cluster.

PAM^a^ level		Low, n (%)	Medium, n (%)	High, n (%)	Very high, n (%)
**Baseline PAM** **level**					
	1	1 (6.3)	2 (6.3)	11 (10.0)	8 (5.1)
	2	2 (12.5)	12 (37.5)	28 (25.5)	42 (26.9)
	3	10 (62.5)	16 (50.0)	56 (50.9)	73 (46.8)
	4	3 (18.7)	2 (6.3)	15 (13.6)	33 (21.2)
**Follow-up PAM level**					
	1	1 (6.3)	3 (9.4)	6 (5.5)	3 (1.9)
	2	0 (0.0)	7 (21.9)	22 (20.0)	31 (19.9)
	3	11 (68.8)	20 (62.5)	63 (57.3)	90 (57.7)
	4	4 (25.0)	2 (6.3)	19 (17.3)	32 (20.5)

^a^PAM: Patient Activation Measure.

**Table 5:** Health care utilization at baseline and follow-up by engagement cluster.

Healthcare utilization type		Low, n (%)	Medium, n (%)	High, n (%)	Very high, n (%)	Total, n (%)
**At least one GP^a^ visit** **in the last 3 months**						
	Baseline	15 (88.2)	28 (84.8)	103 (92.8)	137 (86.2)	283 (88.4)
	Follow-up	14 (82.4)	28 (84.8)	97 (87.4)	129 (81.1)	268 (83.8)
**At least one nurse visit** **in the last 3 months**						
	Baseline	7 (43.8)	6 (21.4)	34 (36.6)	55 (40.7)	102 (37.5)
	Follow-up	5 (31.3)	9 (32.1)	27 (29)	60 (44.4)	101 (37.1)
**At least one Outpatient visit** **in the last 3 months**						
	Baseline	11 (64.7)	16 (51.6)	65 (63.1)	98 (67.6)	190 (64.2)
	Follow-up	9 (52.9)	14 (45.2)	62 (60.2)	85 (58.6)	170 (57.4)
**At least one A&E^b^** **visit** **in the last 3 months**						
	Baseline	2 (11.8)	5 (17.2)	27 (27.8)	28 (19.6)	62 (21.7)
	Follow-up	4 (23.5)	4 (13.8)	18 (18.6)	10 (7)	36 (12.6)
**At least one hospitalization** **in the last 3 months**						
	Baseline	4 (22.2)	7 (21.2)	28 (24.6)	32 (19.6)	71 (21.6)
	Follow-up	1 (5.6)	4 (12.1)	22 (19.3)	24 (14.7)	51 (15.5)

^a^GP: general practitioner.

^b^A&E: accident & emergency.

## Discussion

### Principal Findings

This study found that a group of HU users, who had completed a baseline and follow-up survey, had, on average, a moderate activation score at level 3 *taking action but lacking in confidence*. The improvement in activation over 3 months was, on average, only a modest 2.6 points overall. Overall, 1 in 3 respondents had a lower baseline PAM at levels 1 or 2: this group had the highest change in PAM, with an average increase of 5.8 points, a change thought to be clinically meaningful [14]. There were 4 different levels of engagement with the HU platform (low, medium, high, and very high engagers). Very high engagers used the platform on average 60 days over 3 months, were more frequently female, had no comorbidities, and a diagnosis within the previous year. Their activation increased the most over 3 months. Perhaps, indicating that those most recently diagnosed and with few comorbidities gain the most benefit from high engagement with HU. In terms of health care utilization, overall health care utilization reduced over follow-up. Those who engaged most with HU had fewer visits to A&E at follow-up, although this trend was not seen in other health utilization measures. If these findings represent a causal relationship (see below), it would have important implications for how OHCs can improve outcomes in patients with long-term conditions. Information provision from health care professionals and emerging initiatives such as social prescribing [31] could include directing patients to OHCs. Robust evidence on the effectiveness of OHCs as well as their cost-effectiveness would allow clearer positioning within the armamentarium of treatments for people living with health conditions.

### Representativeness

The study reports on a population of people who completed a baseline survey and a follow-up survey 3 months later. We were able to compare this group with people who completed only the baseline survey and a random sample of other HU users. We noted that survey responders had higher levels of online engagement than those who did not complete the survey. This is perhaps unsurprising as those more motivated to engage with the platform may be more likely to complete the surveys. Nonetheless, this does not detract from comparisons among engagement groups in our study. Thinking further about representativeness, the population for this study was predominately female, older than 50 years, and White. A study of health-related social media users in the United States found similar proportions of health forum users were female [32]; therefore, this population may be a true representation of OHC users in terms of gender. It was expected that users of OHCs would be a younger population, as seen in other studies [6,24,32]; therefore, our older cohort may reflect some selection bias related to willingness to complete surveys. It has been shown that there are still digital disparities in terms of ethnicity, which may reflect why our sample is predominantly White [33]. It has been shown that African Americans had lower PAM levels; these were shown to be mediated through education and health literacy [34,35]. Unfortunately, the numbers are too small to investigate whether PAM was lower in those of non-Caucasian ethnicity in this study.

### Previous Studies

Activation, as measured by the PAM, was similar to a UK sample whose mean PAM score was 59.4. Interestingly, when categorized into levels, only 17% were at level 4 at baseline in this study compared with 21% of a random sample of the UK population, many with chronic conditions [36]. One might have expected patients accessing an OHC to have had higher levels of activation. In a study that examined activation in a Hebrew online social network, the authors found that people who were experienced users of the online social network, classified as those who had used the site for 6 months or more, had significantly higher PAM scores than new users, with a mean PAM 69.3 points for experienced users compared with 62.8 points for new users [24]. Both new and experienced users had higher PAM scores than scores in this study where responders had a mean score of 60.2 and 62.8 points at baseline and follow-up, respectively, although in this study *experienced users* had only used the OHC for 3 months.

### Strengths and Limitations

This was a prospective study of changes to patient activation, health care utilization, and health status with the ability to associate changes with different patterns of engagement with an OHC, with a reasonably large sample size. Meaningful changes in activation were seen in some groups; however, we need to be careful in our interpretation of these findings and must consider some important limitations. First, there was no control group; therefore, we do not know how activation changes in people who did not use an OHC. This makes it difficult to make causal inferences: the small increases in PAM observed in all groups may well be an expected change from the point at which someone signs up to an OHC. It is indeed reasonable to hypothesize that people will sign up at times of greater clinical need. Over the course of the subsequent 3 months, their activation and health care utilization might change for the better regardless of OHC use. Although the clustering allowed identification of those who used HU very little and enabled comparisons across levels of engagement, the numbers were very small, with only 18 and 34 participants in the low and medium engagement groups who completed both baseline and follow-up surveys, making the comparisons across engagement groups less robust. Second, as already mentioned, this was a self-selecting population with only 329 people completing both baseline and follow-up surveys of over 9000 people contacted. This means the sample may not be representative of users of the OHC as a whole, with a skew toward those who engage more with the site. Third, there have been very few longitudinal studies where PAM has been repeatedly measured, which makes it difficult to interpret change in PAM over time. It has been suggested that a change of 5 points is a meaningful difference [14], and we found that 1 in 3 had an increase in PAM score of 5 points or more, although 1 in 10 had a decrease in PAM of 5 or more points. The appropriateness of this threshold for clinical importance is somewhat questionable having been derived from cross-sectional data, where 5 points was identified as the common difference in mean PAM score in people with healthy versus unhealthy behaviors [14]. Fourth, the study’s follow-up was 3 months, which is not very long in terms of disease course and may not be long enough to identify a clear change in activation, health status, or health care utilization. We found a mean increase in PAM of 2.6 points, which is a small change—a longer follow-up may have allowed us to identify a larger change were one to transpire. We were unable to show, despite the small increase in activation, any significant change in health status. This may be because of the length of follow-up. Any interpretation of health status is again hampered by the lack of a control group. In the absence of any engagement with an OHC, it could be argued that health status would either improve (in response to a recent diagnosis and treatment) or worsen (because of progression of disease). We therefore do not know how the OHC engagement has influenced health status. Given these limitations, further investigation is warranted to see how activation changes seen compare with a control population and if certain groups of patients may benefit from OHC use, such as newly diagnosed patients. Understanding what functions within OHCs would deliver better outcomes would also be worthy of future investigation.

### Conclusions

The main findings of this study are that HU users have varied levels of activation when they start using the platform. Patient activation seems to increase over time, although the extent of change did not seem to differ markedly between different levels of platform engagement. Activation increased the most in those with very high engagement with the HU platform and in those with low activation at baseline; however, it is unknown whether these improvements would have been seen irrespective of the use of the platform. Understanding the impact of participation in an OHC on health outcomes will require studies designed specifically to examine this putative causal association.

## Appendix

Multimedia Appendix 1 Exemplar post from HealthUnlocked.

Multimedia Appendix 2 Study survey.

Multimedia Appendix 3 Baseline and follow-up patient activation levels.

Multimedia Appendix 4 3. Box and whisker plot of the number of days of engagement by the number of surveys completed (N=1002).

## Abbreviations

A&E: accident & emergency
EQ-5D: EuroQol-5D
GP: general practitioner
HU: HealthUnlocked
IQR: interquartile range
OHCs: online health communities
PAM: Patient Activation Measure
