# Supplementary material for: Associations Between Engagement With an Online Health Community and Changes in Patient Activation and Health Care Utilization: Longitudinal Web-Based Survey
Source: J Med Internet Res. 2019 Aug 29;21(8):e13477. doi: 10.2196/13477 (PMC6740167; doi:10.2196/13477)
Supplement: Multimedia Appendix 1 [file jmir_v21i8e13477_app1.pdf]

Is angioplasty painful ?

7 days ago • 61 Replies

Hi there I am to have angioplasty in a couple of weeks and would like to know truthfully is it very painful. I was diagnosed with PV JAK2+ about 8 weeks ago (blood cancer) I had a clot in my little toe, so had to see vascular doc, had CT scan never got called back to discuss it, just a letter dropped through my door this morning. I am still reeling from my PV diagnoisis now this I am freaking out.

Related Tags

angioplasty

Reply Like (3) Follow post More

How did this post make you feel?

61 Replies

oldest • newest

7 days ago

I think it can vary.

My Angio + stent was about as uncomfortable as non painful dental work (that's about the nearest I can get to it). Some tugging and pulling in my arm which came up really bruised but which was more uncomfortable than painful and a sensation when the die was released. But nothing too bad and I'd go through it again if I needed to.. and to be honest I'm a bit of a coward when it comes to medical treatment. They give a sedative and I wish I'd asked for more sooner. By the time I asked for more it was all over.

Good luck. I'm sure the Angio will be fine

3 likes • Reply • More

6 days ago

Hi

Dont worry at all.

Believe me it is not painful...of course the usual insertion of the catater in the artery in your arm is similar to giving blood or having a blood test. I had four stents inserted..you get a strange sensation for 30 seconds when the stent is

inserted in place in the artery. ..they warn you . So nothing at all to worry about .best of luck to you..please dont stress

3 likes • Reply • More
