# Supplementary material for: Associations Between Engagement With an Online Health Community and Changes in Patient Activation and Health Care Utilization: Longitudinal Web-Based Survey
Source: J Med Internet Res. 2019 Aug 29;21(8):e13477. doi: 10.2196/13477 (PMC6740167; doi:10.2196/13477)
Supplement: Multimedia Appendix 2 [file jmir_v21i8e13477_app2.pdf]

Activation in Online Peer Support- Feasibility

* **What is your age group?**

- 16-19
- 20-29
- 30-39
- 40-49
- 50-59
- 60-69
- 70 and over

* **What gender best describes you?**

- Male
- Female

What is your current occupation?

- ◯ Student
- ◯ Employed
- ◯ On sick leave, not able to work
- ◯ Unemployed / Looking for work
- ◯ Unemployed and not looking for work (e.g caring for relative/friend)
- ◯ Retired

What is the highest level of education that you have completed?

- ◯ Primary school
- ◯ Secondary school (or equivalent)
- ◯ University degree (or equivalent)
- ◯ Post-graduate degree

Which race/ethnicity best describes you?

- ◯ White / Caucasian
- ◯ Asian
- ◯ Black / African / Caribbean
- ◯ Hispanic / Latino
- ◯ Multiple ethnicity / Other (please specify)

Activation in Online Peer Support- Feasibility

What is your main diagnosis?

____

How long ago were you diagnosed with your condition?

- ◯ Less than a year ago
- ◯ 1 - 3 years
- ◯ 4 - 6 years
- ◯ 7 - 9 years
- ◯ 10 years or more

Do you currently have other long-term health concerns in addition to your primary diagnosis?

- ◯ No
- ◯ Yes, I have 1 more
- ◯ Yes, I have 2 more
- ◯ Yes, I have more than 3

Activation in Online Peer Support- Feasibility

Below are some statements that people sometimes make when they talk about their health.

Please indicate how much you agree or disagree with each statement as it applies to you personally by choosing your answer.

There are no right or wrong answers, just what is true for you. If the statement does not apply to you, choose N/A.

Activation in Online Peer Support- Feasibility

* Please indicate your choice for each statement.

	Disagree Strongly	Disagree	Agree	Agree Strongly	N/A
I am the person who is responsible for taking care of my health	◯	◯	◯	◯	◯
Taking an active role in my own health care is the most important thing that affects my health	◯	◯	◯	◯	◯

Activation in Online Peer Support- Feasibility

* Please indicate your choice for each statement.

	Disagree Strongly	Disagree	Agree	Agree Strongly	N/A
I am confident I can help prevent or reduce problems associated with my health	◯	◯	◯	◯	◯
I know what each of my prescribed medications do	◯	◯	◯	◯	◯
I am confident that I can tell whether I need to go to the doctor or whether I can take care of a health problem myself	◯	◯	◯	◯	◯

Activation in Online Peer Support- Feasibility

* Please indicate your choice for each statement.

	Disagree Strongly	Disagree	Agree	Agree Strongly	N/A
I am confident that I can tell a doctor or nurse concerns I have even when he or she does not ask	○	○	○	○	○
I am confident that I can carry out medical treatments I may need to do at home	○	○	○	○	○
I understand my health problems and what causes them	○	○	○	○	○

Activation in Online Peer Support- Feasibility

* Please indicate your choice for each statement.

	Disagree Strongly	Disagree	Agree	Agree Strongly	N/A
I know what treatments are available for my health problems	○	○	○	○	○
I have been able to maintain lifestyle changes, like healthy eating or exercising	○	○	○	○	○
I know how to prevent problems with my health	○	○	○	○	○

Activation in Online Peer Support- Feasibility

* Please indicate your choice for each statement.

	Disagree Strongly	Disagree	Agree	Agree Strongly	N/A
I am confident I can work out solutions when new problems arise with my health	○	○	○	○	○
I am confident that I can maintain lifestyle changes, like healthy eating or exercising, even during times of stress	○	○	○	○	○

Activation in Online Peer Support- Feasibility

By selecting one option in each category, please indicate which statements best describe your own health state today.

Mobility

- ◯ I have no problems in walking about
- ◯ I have slight problems in walking about
- ◯ I have moderate problems in walking about
- ◯ I have severe problems in walking about
- ◯ I am unable to walk about

Self-Care

- ◯ I have no problems washing or dressing myself
- ◯ I have slight problems washing or dressing myself
- ◯ I have moderate problems washing or dressing myself
- ◯ I have severe problems washing or dressing myself
- ◯ I am unable to wash or dress myself

Usual Activities (e.g. work, study, housework, family or leisure activities)

- ◯ I have no problems doing my usual activities
- ◯ I have slight problems doing my usual activities
- ◯ I have moderate problems doing my usual activities
- ◯ I have severe problems doing my usual activities
- ◯ I am unable to do my usual activities

Pain / Discomfort

- ◯ I have no pain or discomfort
- ◯ I have slight pain or discomfort
- ◯ I have moderate pain or discomfort
- ◯ I have severe pain or discomfort
- ◯ I have extreme pain or discomfort

Anxiety / Depression

- ◯ I am not anxious or depressed
- ◯ I am slightly anxious or depressed
- ◯ I am moderately anxious or depressed
- ◯ I am severely anxious or depressed
- ◯ I am extremely anxious or depressed

In the last 3 months, roughly how many times have you visited the following health care services?

	Number of visits
GP	
Outpatient clinic	
Primary care nurse	
Accidents & Emergency (A&E) department	

In the last 3 months, roughly how many days would you have spent admitted in a hospital?

Activation in Online Peer Support- Feasibility

Prior to visiting HealthUnlocked, did you know anyone else in a similar health situation offline?

- ◯ Yes
- ◯ No

Which of the following best describes what you are looking for on the HealthUnlocked platform?

- ◯ Connect with people similar to you through communities
- ◯ Know more about your health condition through posts and understand treatments better
- ◯ Manage your symptoms and health conditions
- ◯ Help people with similar health conditions by sharing your experiences

Activation in Online Peer Support- Feasibility

* Please provide your contact number where you would like to be reached.

We encourage you to share your mobile number with us to help us reach you via SMS for a follow-up survey, only if you do not respond to our mail.

Please note that despite providing your contact information, no names or identifying information will be included in any publications or presentations and your responses will remain confidential.

Where do you reside?

Country

Thank you!

We will contact you again for a follow-up survey in a few months.
