# Supplementary material for: Associations Between Engagement With an Online Health Community and Changes in Patient Activation and Health Care Utilization: Longitudinal Web-Based Survey
Source: J Med Internet Res. 2019 Aug 29;21(8):e13477. doi: 10.2196/13477 (PMC6740167; doi:10.2196/13477)
Supplement: Multimedia Appendix 3 [file jmir_v21i8e13477_app3.pdf]

Baseline and follow-up patient activation levels

		Baseline PAM				
		1	2	3	4	Total N (Column %)
Follow-up PAM N (row %)	1	9 (2.9)	4 (1.3)	0 (0)	0 (0)	13 (4.1)
	2	6 (1.9)	34 (10.8)	17 (5.4)	3 (1)	60 (19.1)
	3	7 (2.2)	45 (14.3)	113 (36)	19 (6.1)	184 (58.6)
	4	0 (0)	1 (0.3)	25 (8)	31 (9.9)	57 (18.2)
	Total	22 (7)	84 (26.8)	155 (49.4)	53 (16.9)	314 (100)

Shading key: green = increased PAM level, white = stable PAM level, red = decreased PAM level
