# Supplementary figures and images for: Associations Between Engagement With an Online Health Community and Changes in Patient Activation and Health Care Utilization: Longitudinal Web-Based Survey
Source: J Med Internet Res. 2019 Aug 29;21(8):e13477. doi: 10.2196/13477 (PMC6740167; doi:10.2196/13477)

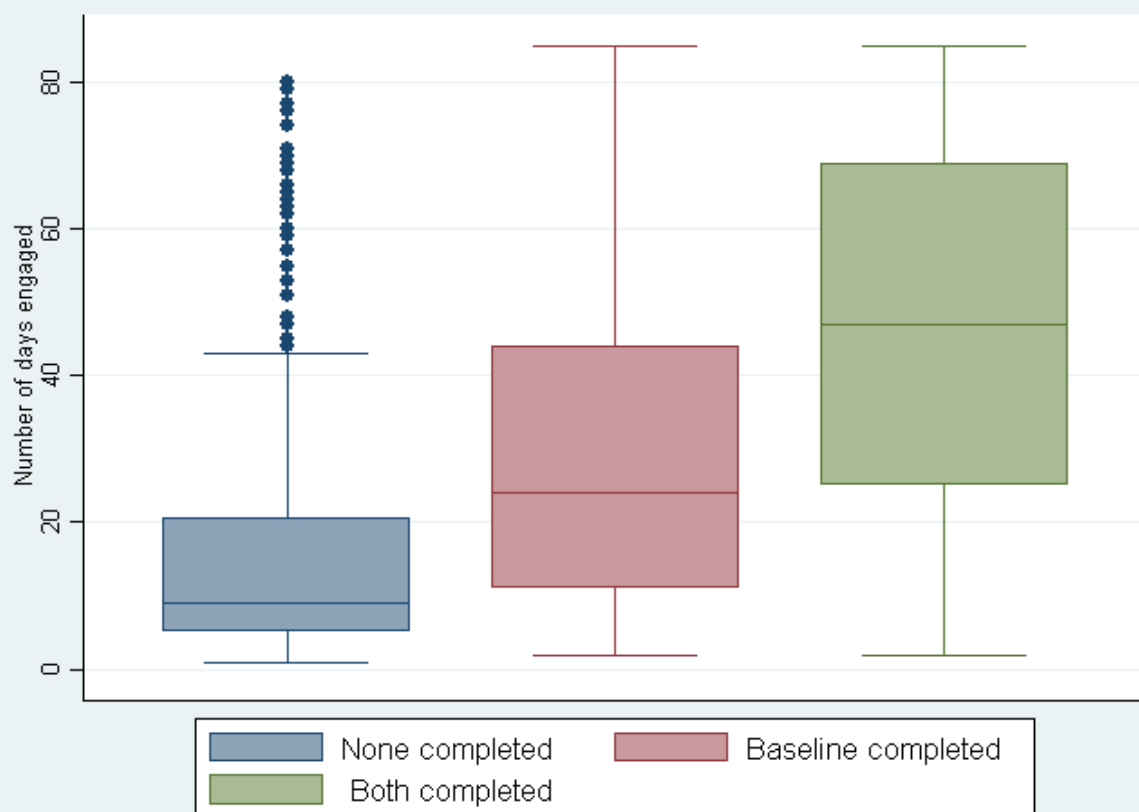

Supplement: Multimedia Appendix 4 [file jmir_v21i8e13477_app4.pdf]
